# Mental Training and Flow in Wheelchair Basketball: The Mediating Role of Injury Anxiety

**DOI:** 10.3390/healthcare13222944

**Published:** 2025-11-17

**Authors:** Mehdi Duyan, Talip Çelik, İlker Günel, Gülcan Tekin, Ali Tekin, Fatma Özoğlu, Mihriay Musa, Emrah Barkın

**Affiliations:** 1 Faculty of Sports Sciences, İnönü University, 44280 Malatya, Türkiye; fatma.ozoglu@inonu.edu.tr; 2 Malatya Vocational School, İnönü University, 44000 Malatya, Türkiye; 3 School of Physical Education and Sport, Osmaniye Korkut Ata University, 80000 Osmaniye, Türkiye; kergunel@gmail.com; 4 Faculty of Sports Sciences, Agri İbrahim Çeçen University, 04100 Agri, Türkiye; gtekin@agri.edu.tr (G.T.); alitekin@agri.edu.tr (A.T.); 5 Faculty of Sports Sciences, Topkapı University, 34010 Istanbul, Türkiye; mihriaymusa@topkapi.edu.tr; 6 Academy Private Education Institution, 35410 Izmir, Türkiye; brknemrah@gmail.com

**Keywords:** mental training skills, injury anxiety, flow, optimal performance, wheelchair basketball

## Abstract

Background/Objectives: The current study aimed to investigate the mediating role of injury anxiety in the relationship between mental training and flow state among wheelchair basketball athletes. Although mental training is known to be essential for enhancing athletic performance, the underlying mechanisms through which it affects psychological states such as flow have been studied very limitedly, especially in adaptive sports. Methods: The research was conducted using a correlational survey model within a quantitative research framework. The study sample consisted of a total of 153 elite male wheelchair basketball athletes competing in leagues in Türkiye. Hayes’ Process Macro Model was used to test the mediating effect in the analysis of the data. Results: The analyses revealed that mental training significantly reduced injury anxiety (*β* = −0.328, *p* < 0.001), accounting for 7.6% of its variance. Injury anxiety had a significant negative effect on optimal performance (*β* = −0.3380, *p* < 0.001). The direct effect of mental training on performance remained positive and significant (*β* = 0.4324, *p* < 0.001). Together, the variables explained 43% of the variance in optimal performance. The total effect of mental training was also significant (*β* = 0.543, *p* < 0.001). Conclusions: In conclusion, the findings of this study reveal that mental training plays a significant role in both reducing injury anxiety and enhancing optimal performance among wheelchair basketball players. It was determined that injury anxiety functions as a mediating variable in the relationship between mental training and flow.

## 1. Introduction

The concept of flow is defined as a positive experience associated with optimal performance and psychological well-being, which is increasingly studied in the field of exercise and sports psychology [1,2]. According to Csikszentmihalyi [3], who introduced the concept, flow is a state of complete immersion in an activity characterized by intense focus, loss of self-awareness, and an altered perception of time and effort. In athletes, flow is consistently associated with enhanced attention and concentration, increased self-confidence, and high-level performance [4,5]. Since athletic success is often linked to the ability to effectively focus and sustain attention [6], understanding the factors that facilitate flow states in sports settings is considered one of the fundamental goals of exercise and sports psychology.

However, reaching a state of flow can be threatened by psychological barriers such as fear of injury. This is particularly evident in high-risk sports such as wheelchair basketball. Wheelchair basketball is a Paralympic team sport played between two teams of five physically disabled athletes [7,8]. Consequently, wheelchair basketball requires extensive upper limb movements (shooting, catching, passing, etc.) and involves high contact (wheelchair collisions, etc.), which leads to a high incidence of sports injuries among players [8,9]. Injury anxiety and its associated kinesiophobia (fear of movement) [10,11] are defined as the fear of injury or re-injury. This anxiety distracts the athlete’s attention from the performance task and directly conflicts with complete focus, which is a fundamental prerequisite for flow state [12,13]. Recent studies emphasize that fear of injury is not only an individual source of stress, but also deeply embedded in the athlete’s social environment (team dynamics, coach relationships, etc.) and can seriously disrupt the focus and automatic processing necessary for optimal performance [14].

For example, in a 44-week prospective study conducted with elite Swedish para-athletes, Bentzen et al. [15] found that 15% of athletes exhibited anxiety and 21% exhibited depressive symptoms in the initial weekly observations, and that these rates rose to 58% and 42%, respectively, by the end of the study. Similarly, Juggath and Naidoo [16] observed that approximately 60% of athletes experienced injury anxiety following a sports injury, which negatively affected both their performance and psychological state. Injury anxiety has also been shown to be associated with loss of motivation and various other psychological responses [17]. Therefore, mental training skills play an important role in this situation. Mental training interventions aimed at developing psychological skills are suggested as a potential way to reduce injury anxiety [18]. Research shows that mental training has positive effects on athletes’ fundamental psychological skills such as stress management, mental resilience, emotional regulation, self-confidence, attention, and concentration [19,20,21,22,23,24]. The need to consider the relationship between psychological stress, anxiety, and musculoskeletal problems in sports injuries is also emphasized [25,26], and a study synthesizing 50 years of evidence confirms the profound impact of psychological factors on both injury risk and the rehabilitation process [27].

However, while current research indicates that Paralympic athletes face unique challenges affecting their psychological health [28], and a positive relationship between sports participation and psychological well-being and quality of life has been established in individuals with disabilities [29,30], the specific psychological needs of this population related to performance optimization have not been sufficiently investigated.

Research comprehensively examining the dynamic relationships between mental training skills, injury anxiety, and flow state, particularly in the context of wheelchair basketball, remains significantly limited. More importantly, how mental training develops flow—particularly the mediating role of a fundamental psychological barrier such as injury anxiety in this relationship—remains unclear. Bernier et al.’s [31] call to test theoretical models revealing change mechanisms, combined with Bingöl et al.’s [10] emphasis on the need to address injury anxiety in physically disabled athletes, guided the purpose of this study. Therefore, the purpose of this study is to examine whether injury anxiety plays a mediating role in the relationship between mental training skills and flow state in wheelchair basketball players. The hypotheses developed in line with this purpose are stated below:

**H_1_:**  *Mental training skills positively and significantly affect the flow of wheelchair basketball players.*

**H_2_:**  *Mental training leads to a significant reduction in wheelchair basketball players’ injury anxiety.*

**H_3_:**  *Injury anxiety negatively and significantly affects wheelchair basketball players’ flow.*

**H_4_:**  *Injury anxiety plays a mediating role in the relationship between mental training skills and flow.*

**Figure 1:**
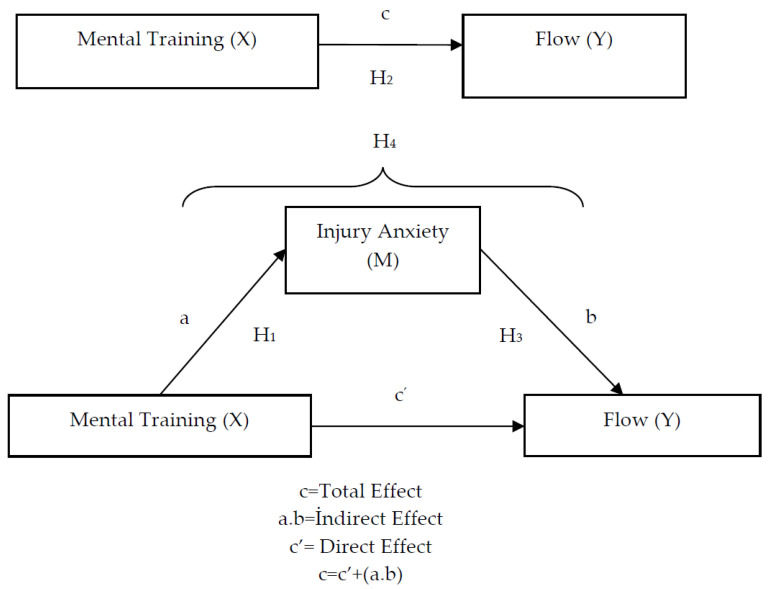
Mediation model.

In summary, the hypotheses developed to examine the relationship between mental training, flow and injury anxiety behavior using a mediation model are presented in Figure 1.

## 2. Materials and Methods

In this study, a relational screening model was used as one of the quantitative research methods. The relational screening model is a research model that aims to determine the level of variability and relationship between two or more variables [32].

### 2.1. Research Design

The present study utilized a correlational research design to examine the potential mediating role of injury anxiety in the relationship between mental training and flow state. The hypothesized mediation model is presented in Figure 1.

The model illustrates three key paths:

1. The effect of the independent variable (mental training) on the mediator variable (injury anxiety), denoted as Path a.
2. The effect of the independent variable (mental training) on the dependent variable (flow state), denoted as Path c’ (the direct effect).
3. The effect of the mediator variable (injury anxiety) on the dependent variable (flow state), denoted as Path b.

The primary objective of mediation analysis within the contemporary approach is to calculate and test the significance of the indirect effect. This indirect effect is quantified by the product of the coefficients from Path a and Path b (i.e., a × b). According to this approach, if bootstrap analysis (with 5000 samples) for the model in Figure 1 reveals a significant indirect effect (where the 95% bias-corrected confidence interval does not include zero), the mediating role of injury anxiety is considered statistically supported [33].

A model of relationships among mental training, flow, and injury anxiety.

### 2.2. Sample Size and Procedure

Considering the factors of time, accessibility and cost, the sample selection was carried out using the convenience sampling method. This method is a practical sampling technique frequently used in the social sciences, offering researchers the opportunity to reach participants quickly and effectively [34]. Within the scope of the research, a prior power analysis was conducted using G*Power 3.1.9.7 software to determine the sample size. Considering the three predictor variables in the structural model, the analysis parameters were set as follows: 5% significance level (α = 0.05), 95% test power (1 − β = 0.95), and a small effect size (f^2^ = 0.15) as per Cohen’s [35] conventions. Accordingly, the minimum sample size required for the study to produce valid results was calculated to be 119 individuals.

The inclusion criteria for the study were as follows: (a) being aged 18 or over, (b) actively playing licensed wheelchair basketball in the Regional League, First Division, Second Division, or Super League within the men’s league in Türkiye, and (c) volunteering to participate. Data on the specific etiology or medical diagnosis of the participants’ physical disabilities (e.g., spinal cord injury, cerebral palsy, limb deficiency) were not collected, as the study focused on their functional status as competitive wheelchair athletes.

Conversely, the exclusion criteria were defined as: (a) playing in the women’s wheelchair league, (b) declining to participate in the study, (c) having a history of serious injury in the last three months, and (d) being unable to complete the assessment due to health problems that could affect physical or cognitive functioning.

Data were collected via questionnaire forms shared with participants through online communication tools. Although a total of 158 forms were returned, 5 forms containing extreme outliers were excluded from the analysis, leaving 153 valid data forms. A total of 153 male athletes meeting these criteria were included in the study. Summary information regarding the participants’ demographic characteristics is presented in Table 1.

### 2.3. Data Analysis

Data analysis was conducted using SPSS 25 (IBM, Armonk, NY, USA) and LISREL 8.70 (Scientific Software International, Inc., Skokie, IL, USA) software packages, along with the Process macro developed by Hayes [36]. Prior to hypothesis testing, preliminary analyses were performed to screen for missing data, outliers, and to assess the assumption of normality. A Confirmatory Factor Analysis (CFA) was conducted using LISREL 8.70 to validate the factor structure of the scales. The normality of the data distribution for the main study variables was confirmed using the Kolmogorov–Smirnov test in SPSS, yielding the following results: mental training scale (0.082, *p* > 0.05), flow state scale (0.076, *p* > 0.05), and injury anxiety scale (0.091, *p* > 0.05).

The internal consistency of the scales was assessed using Cronbach’s alpha. The reliability coefficients were interpreted based on the following standard thresholds [37,38]: ≥0.90 (Excellent), ≥0.80 (Good), ≥0.70 (Acceptable), ≥0.60 (Questionable), and <0.60 (Poor). The strength of correlation coefficients (r) was interpreted according to Cohen’s [35] conventions, where r ≥ 0.50 is considered large, r = 0.30 to 0.49 medium, and r = 0.10 to 0.29 small.

Descriptive statistics, including means, standard deviations (SD), and percentages (%), were calculated. Pearson correlation analysis was employed to examine the bivariate relationships between the variables. To test the proposed mediation model—specifically, the relationship between mental training and flow state and the mediating role of injury anxiety—a regression-based mediation analysis was performed using the Process macro (Model 4) with the bootstrap method. This analysis utilized 5000 bootstrap samples, and the significance of the indirect effect was determined by a 95% bias-corrected confidence interval. The a priori significance level (alpha) was set at *p* < 0.05 for all statistical tests. In the interpretation and reporting of results, a more conservative threshold of *p* < 0.01 was also applied to identify highly robust effects, given the study’s sufficient statistical power.

### 2.4. Self-Report Measures

#### 2.4.1. Mental Training Scale (MTS)

In this study, the “Mental Training Scale” developed by Behnke et al. [39] and adapted into Turkish by Yarayan and İlhan [40] was used to assess athletes’ mental skills and techniques. The scale consists of 20 items and five subscales (Mental Basic Skills, Mental Performance Skills, Interpersonal Skills, Self-Talk, Mental Imagery) and is scored using a 5-point Likert scale. According to the confirmatory factor analysis reported in the Turkish adaptation study [40], the scale demonstrated an acceptable level of model fit, as indicated by several key indices: χ^2^/df = 1.85, GFI = 0.91, CFI = 0.95, NFI = 0.91, and AGFI = 0.88. The scale’s validity was further supported by high internal consistency coefficients, ranging from 0.82 to 0.91 [40], which are classified as “Good” to “Excellent” according to standard reliability thresholds [37,38].

#### 2.4.2. Sports Injury Anxiety Scale (SIAS)

In this study, the Sports Injury Anxiety Scale (SIAS) developed by Rex and Metzler [41] and adapted into Turkish by Caz et al. [42] was used to measure injury anxiety in athletes. The scale is structured as a five-point Likert scale and consists of 19 items grouped into six subscales: fear of losing ability, fear of being perceived as weak, fear of pain, fear of disappointment, fear of losing social support, and fear of re-injury. The Turkish adaptation of the scale has demonstrated strong psychometric properties. The original adaptation study [42] confirmed the six-factor structure and 9 reported excellent internal consistency for the total score (α = 0.870), with subscale reliabilities ranging from 0.608 to 0.876. In line with the aim of our study, the total score of the scale was utilized for data analysis.

#### 2.4.3. Dispositional Flow Scale-2 (DFS-2)

The short form developed by Jackson and Eklund [43], consisting of 9 items, was used in this study. The Turkish adaptation of the scale was performed by Çağlar et al. [44]. The psychometric evaluation conducted by the same researchers demonstrated that the scale is a valid and reliable instrument. Confirmatory factor analysis showed acceptable fit indices (χ^2^ = 93.242, df = 27, SRMR = 0.06, CFI = 0.92, RMSEA = 0.08, NNFI = 0.88), while the composite reliability coefficient (0.78) and Cronbach’s alpha internal consistency coefficient (0.77) were found to be at adequate levels [45], which meet the “acceptable” threshold according to standard reliability criteria [37,38].

## 3. Results

### 3.1. Descriptive Statistics

The study sample consisted of 153 male wheelchair basketball players. The mean age of the players was 28.4 ± 1.27 years, and it was determined that 47.7% of the players were aged 30 and above. When examined according to educational level, 43.8% of the players were high school graduates. 60.1% of players stated that they became disabled later in life. In terms of sporting background, 50.3% of players had a total of 9 years or more of sporting experience. Their distribution by league level was as follows: Regional League (14.4%), 1st League (23.5%), 2nd League (32%), and Super League (30.1%). When the responses to the question ‘body region where you suffered an injury’ were examined, it was found that 56.1% suffered injuries to the head region and 23.5% to the torso region. Detailed findings regarding the demographic and sporting characteristics of the players are presented in Table 1.

### 3.2. Validity and Reliability Analysis

According to the results of the confirmatory factor analysis conducted to test construct validity, the X^2^/df ratios of the scales and the values of other fit indices were found to be within acceptable limits [37,38]. In addition, the examination of Cronbach’s alpha coefficients indicated that the mental training, flow, and injury anxiety scales demonstrated a high level of reliability [37,38] (Table 2).

### 3.3. Correlation Analyses

The results of the Pearson correlation analyses revealed significant relationships between mental training, injury anxiety and optimal performance variables within the research model (Table 3).

The results of the correlation analysis conducted in this study revealed a low, negative, and significant relationship between mental training and injury anxiety (r = −0.277; *p* < 0.01). In contrast, a moderate and positive significant relationship was found between mental training and flow (r = 0.53; *p* < 0.01). Furthermore, a moderate, negative, and significant relationship was established between injury anxiety and flow (r = −0.51; *p* < 0.01). The strength of the correlation coefficients was interpreted using conventional benchmarks (small 0.10, medium 0.30, large 0.50) [37,38]. The findings indicate that mental training may play a role in enhancing flow and reducing injury anxiety. Furthermore, it appears that reduced injury anxiety enhances flow, which is optimal performance.

### 3.4. Bootstrap Regression Analysis

In this study, the mediation analysis examining the effect of mental training on optimal performance and injury anxiety was conducted using a contemporary approach in line with the criticisms leveled at Baron and Kenny’s traditional method. In the contemporary approach, the mediating effect is tested using the bootstrap technique, which yields stronger and more reliable results than the Sobel test. The indirect effect was calculated by multiplying the effect of the independent variable (mental training) on the mediating variable (injury anxiety) (path a) by the effect of the mediating variable on the dependent variable (optimal performance) (path b). Using the bootstrap method, 5000 repetitions were performed, and when the values obtained within the 95% confidence interval did not include zero, the mediating effect was considered significant. The analyses were conducted using the Process macro developed by Hayes [36]. The findings obtained from the regression analysis conducted for this purpose are presented in Table 4.

The hypotheses developed within the scope of the mediation test were analyzed. Firstly, the regression analysis findings revealed the effect of mental training (X) on the mediating variable of injury anxiety (M) (Path a). According to Table 4, mental training significantly and negatively affects injury anxiety (β = −0.328, t = −3.72, *p* < 0.001). The significance level being below 0.001 supports this result. Accordingly, mental training explains approximately 7.7% of the variation in injury anxiety (R^2^ = 0.076).

Subsequently, the combined effect of the mediator variable injury anxiety (M) (Path b) and the direct effect of mental training (X) (Path c’) on flow state (Y) was tested. The findings indicate that injury anxiety has a statistically significant and negative effect on flow state (β = −0.338, t = −6.49, *p* < 0.001). The direct effect of mental training was found to be significant and positive (β = 0.432, t = 7.00, *p* < 0.001). Mental training and injury anxiety together explain approximately 43.2% of the variation in flow state (R^2^ = 0.432). Furthermore, when the mediator variable was not included in the model (Path c = total effect), the effect of mental training on flow state was found to be statistically significant (β = 0.543, R^2^ = 0.287, t = 8.20, *p* < 0.001).

In the final stage, the indirect effect of mental training on flow state through the mediating variable of injury anxiety was examined using the Bootstrap regression model with 5000 samples. The results presented in Table 4 show that the indirect effect is statistically significant (β = 0.111, *p* = 0.002). The 95% confidence interval [0.044, 0.186] does not include zero, indicating that injury anxiety mediates the relationship between mental training and flow state.

**Figure 2:**
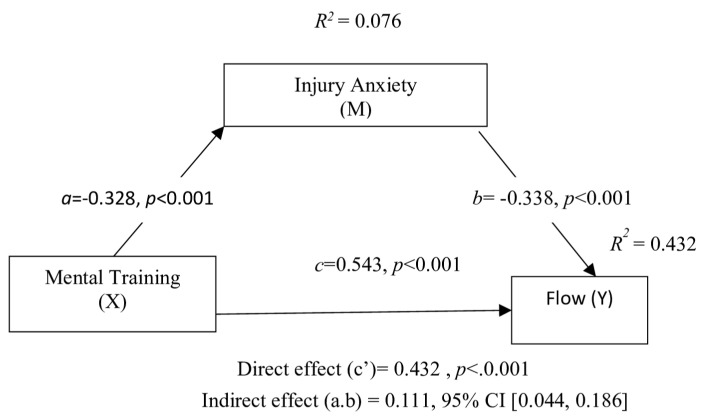
Display of analysis results on model.

The fully standardized effect size (K^2^) was calculated as 0.109, with a 95% confidence interval [0.045, 0.176]. Following the guidelines proposed by Hayes [36] for interpreting mediation effect sizes, where K^2^ values around 0.01 indicate a small effect, 0.09 a medium effect, and 0.25 a large effect, the mediation effect in our model can be interpreted as medium. Consequently, all four hypotheses of the study have been supported. The results of the mediation analysis are presented graphically in Figure 2.

## 4. Discussion

This study included only male athletes to maintain methodological consistency. The literature shows significant gender differences in anxiety levels and coping mechanisms. Including female athletes could have obscured the specific relationship between mental training, injury anxiety, and flow that we aimed to investigate. Furthermore, the limited number of elite female wheelchair basketball players in Türkiye presented a practical constraint. While this focused approach strengthens the validity of our findings for male athletes, it limits the generalizability of our results to female players, which should be explored in future research.

### 4.1. The Relationship Between Mental Training and Flow

The strong positive relationship between mental training and flow state (β = 0.432, *p* < 0.001) supports existing literature on the efficacy of psychological skills training in enhancing athletic performance. The results indicate that mental training has a strong positive relationship with athletes’ flow state. Mental training practices can be said to help athletes maintain emotional balance before and during competition, cope with stress, and maintain concentration [2]. One reason for this is that mental training techniques (e.g., imagery, self-efficacy training, breath control) indirectly contribute to athletes’ performance by increasing their intrinsic motivation [46]. For athletes competing under high pressure, these practices trigger positive emotional states and strengthen mental resilience. Furthermore, mental training enhances not only the sense of achievement but also athletes’ self-confidence and emotional satisfaction, making the performance experience more meaningful [47]. Mental training has positive effects on athletes’ optimal performance by reducing stress and anxiety [48,49]. A 10-week mindfulness-based intervention program (including flow meditation) was found to improve the ability of individual and team athletes to cope with high levels of stress in daily life. The program also helped athletes cope more effectively with factors such as fear of failure that arise in intensely competitive environments and manage competition in a healthier way [50]. In mental training programs, it is stated that the development of body awareness, attention regulation and self-regulation skills is important in gaining the psychological skills necessary for athletes’ performance [51].

### 4.2. The Relationship Between Mental Training and Athletes’ Injury Anxiety

The significant reduction in injury anxiety through mental training (β = −0.328, *p* < 0.001) demonstrates the protective role of psychological skills in managing sport-related anxiety. These results are consistent with the existing literature, which shows that mental training and mindfulness-based interventions are particularly effective in reducing competition anxiety and managing fear of re-injury among athletes [52,53,54,55]. The current literature indicates that athletes returning to sport before completing their mental preparation process may experience problems such as intense anxiety, stress, risk of re-injury, depressive symptoms, and decline in performance [56,57,58,59]. Our conclusions are consistent with current research. For instance, Akın et al. [60] found that a 10-week mental training program for young footballers resulted in a significant decrease in athletes’ injury anxiety levels and a significant increase in their stress coping skills. However, there are findings that do not support our study. Kaplan and Andre [61] found no relationship between mental training status and injury anxiety in athletes involved in team or individual sports. A recent and comprehensive meta-analysis by Li et al. [62] synthesized findings from controlled studies and provides evidence that psychological interventions reduce anxiety levels in athletes. It has been stated that lack of self-confidence and inadequate attention and emotional control skills increase injury anxiety in team athletes; mental preparation supported by psychological interventions is an effective method to reduce this anxiety [18]. Psychological skills training helps reduce the anxiety caused by negative emotions by improving athletes’ ability to regulate and control their reactions to events [20,63]. Consequently, athletes can more effectively cope with stressors that arise before and during competition, maintain focus, and optimize their performance [64]. Furthermore, regular psychological skills training has been shown to develop emotional resilience and mental resilience in athletes, which in turn contributes positively to post-injury recovery [65].

### 4.3. The Relationship Between Injury Anxiety and Flow

The negative relationship between injury anxiety and flow (β = −0.338, *p* < 0.001) underscores the cognitive interference mechanism through which anxiety impairs performance. The findings of this study reveal that a reduction in injury anxiety among athletes significantly increases their level of optimal performance. Injury anxiety is not limited to the perception of physical risk; it also has negative effects on athletes’ attention levels, decision-making abilities, and self-confidence. Other studies have reached similar conclusions. A prospective cohort study of runners with lower-body injuries revealed that athletes with high anxiety levels had significantly longer recovery times (7.9 ± 4.1 months vs. 5.0 ± 3.1 months) and less optimized biomechanical development compared to their low-anxiety peers [66]. A study shows that poor attentional control in young volleyball players negatively impacts dynamic balance performance and may increase the risk of injury. Balance control, particularly in the non-dominant leg, has been found to be directly related to neurocognitive processes [67]. Research findings show that athletes who have been injured before experience injury anxiety, which leads to motivational losses [68]. It is observed that anxiety levels increase, self-confidence decreases and avoidance behaviors develop in athletes experiencing kinesiophobia (fear of movement) [11].

### 4.4. The Mediating Effect of Injury Anxiety: Theoretical Implications

The significant mediation effect (indirect effect = 0.111, 95% CI [0.0449, 0.1860]) provides empirical support for a sequential psychological pathway in which mental training first reduces injury anxiety, which in turn facilitates flow states. One of the most noteworthy findings of this study is that it reveals injury anxiety plays a mediating role in the relationship between mental training and optimal performance. This psychological process is particularly critical for athletes with physical disabilities. Because they face a higher risk of injury, managing this anxiety through mental training is considered crucial for achieving flow and optimal performance [69].

Given their increased susceptibility to injury, the ability to manage injury-related anxiety through mental training becomes crucial for achieving optimal performance states. In this context, mental training skills are proving effective not only in reducing injury anxiety but also in fostering focused emotional engagement in sport. Research suggests that mindfulness-based interventions are particularly valuable in this context, effectively supporting psychological well-being and performance by addressing injury anxiety during return-to-sport processes [70,71,72].

To clarify the reasons behind this mediation model, the underlying psychological mechanisms can be examined using established theoretical frameworks. The effect of mental training on reducing injury anxiety is consistent with the principles of Cognitive-Behavioral Theory [73]. This theory proposes that mental training enables athletes to recognize and change dysfunctional thought patterns, thereby achieving cognitive restructuring. Subsequently, the negative relationship between injury anxiety and flow can be explained by Attention Control Theory [74]. According to this theory, anxiety diverts attention away from the task and toward internal threats, which consumes limited mental resources. As a result, the absolute task focus that enables flow is disrupted.

A holistic view of this entire process can be provided by the Broaden-and-Build Theory [75]. This theory proposes that positive psychological interventions, such as mental training, enable the athlete to escape the constricting effects of injury anxiety by “broadening” their repertoire of thoughts and actions. This mental expansion creates the space necessary for the “building” of a positive and productive psychological state such as flow. Ultimately, the mediating effect reveals a clear psychological process: Mental training alleviates anxiety and expands mental capacity through cognitive restructuring; this, in turn, frees up attention resources to be used for intense focus in a state of flow.

Consistent with these findings, Weiß et al. [76] emphasize that psychological interventions serve as performance-enhancing factors both during post-injury rehabilitation and for athletes struggling with injury anxiety. Therefore, mental training can be considered an effective approach for promoting optimal performance states through anxiety reduction, particularly among athletes with physical disabilities.

### 4.5. Practical Implications for Sport Psychology Practice

The demonstrated mediation effect suggests that interventions targeting injury anxiety may amplify the benefits of mental training for flow enhancement. This finding supports the strategic integration of evidence-based anxiety management techniques-such as mindfulness, cognitive restructuring, and exposure training-into mental skills programs for wheelchair athletes [53,54,77]. The 43% variance explained in flow state indicates that combining mental training with specific anxiety-reduction strategies could substantially improve intervention effectiveness.

Collectively, these findings suggest that mental training skills may serve as an effective method for reducing injury anxiety in athletes. First, by reducing injury anxiety, these skills allow athletes to feel more confident in the sport environment and focus more easily on their training. Second, mental training skills have positive effects on athletes’ fundamental psychological skills, such as stress management, emotional regulation, self-confidence, attention, and concentration. Athletes can better manage their emotions in challenging situations and minimize the negative effects of stress on performance. Furthermore, thanks to their increased self-confidence, athletes become more confident in their abilities and can maximize their concentration during competition by eliminating distractions. Finally, mental training not only reduces injury anxiety but also increases athletes’ overall psychological resilience, contributing significantly to both training efficiency and competitive performance. Therefore, systematically integrating mental training programs into athletes’ development is considered critical for both psychological resilience and performance optimization.

### 4.6. Limitations and Future Research Directions

The findings of this study are subject to several methodological limitations. First, the exclusive focus on male wheelchair basketball players restricts the generalizability of the results to other disability groups, sports disciplines, and female athletes. This sampling strategy was implemented primarily for methodological consistency, as established gender differences in psychological constructs such as injury perception and coping strategies could confound the specific relationships under investigation. The limited population size of elite female wheelchair basketball players in Türkiye also presented a practical constraint in forming a robust comparative sample within the study’s scope. Secondly, while the study focused on athletes with physical disabilities, the specific medical diagnoses (e.g., spinal cord injury, cerebral palsy) were not recorded. Future research could incorporate detailed medical classification to explore potential variations in psychological outcomes across different disability etiologies. Third, participants were not screened for pre-existing psychological conditions such as clinical anxiety, depression, or post-traumatic stress disorder. The presence of such conditions may have influenced baseline levels of the psychological variables measured, representing a potential confounding factor. Furthermore, the use of a homogeneous sample in terms of both sport type and disability category may limit the applicability of the findings across different adaptive sports contexts. Additionally, this study examined the athlete group as a whole and did not investigate potential differences based on the timing of disability acquisition (e.g., congenital versus acquired). This factor may influence psychological variables such as injury anxiety and represents an important avenue for future research.

A cross-sectional research design was employed in this study, and there is a need for longitudinal or experimental designs in the future to allow for causal inferences. Additionally, other potential psychological mediators such as self-efficacy, motivation, coping strategies, or levels of social support were not considered in the study.

The following recommendations can be offered for future research: (1) the use of longitudinal research designs to test causal relationships, (2) the utilization of multiple data sources such as coach evaluations and performance data, (3) conducting studies with diverse samples including different disability groups, sports disciplines, and gender distributions, (4) the inclusion of detailed disability classification to better account for population heterogeneity, and (5) the integration of wearable technologies into research designs to objectively monitor stress and anxiety management processes.

## 5. Conclusions

This study presents a comprehensive framework by examining the relationships between mental training, injury anxiety, and flow state in a sample of male wheelchair basketball players. The findings reveal that (1) mental training has significant positive effects in both reducing injury anxiety and enhancing athletes’ flow experiences. Psychological skills training provides athletes with the tools to manage emotional states and maintain focus under competitive pressure. (2) Injury anxiety was found to mediate the relationship between mental training and flow state, clearly demonstrating how psychological barriers can impact optimal performance experiences. The reduction in injury-related concerns creates a more conducive environment for flow occurrence. (3) Particularly for athletes with disabilities, mental training functions not only as a performance-enhancing factor but also as an anxiety-regulating element. In this context, the development of mental skills plays a critical role in building psychological resilience and confidence necessary for flow states.

## Figures and Tables

**Table 1:** Demographic characteristics of athletes.

Variables		f	%
Marital Status	Single	104	68.0
	Married	49	32.0
	Total	153	100.0
Age	18–21 years old	40	26.1
	22–25 years old	17	11.1
	26–29 years old	23	15.0
	30 years old and above	73	47.7
	Total	153	100.0
Education Status	Primary/Secondary Education	21	13.7
	High School	67	43.8
	University	65	42.5
	Total	153	100.0
Time of Disability	Congenital	61	39.9
	Acquired	92	60.1
	Total	153	100.0
League Represented	Regional League	22	14.4
	1st League	36	23.5
	2nd League	49	32.0
	Premier League	46	30.1
	Total	153	100.0
Total Sports Experience	1–2 years	19	12.4
	3–4 years	19	12.4
	5–6 years	20	13.1
	7–8 years	18	11.8
	9 years and above	77	50.3
	Total	153	100.0
Body part where you suffered an injury	Head	79	51.6
	Torso	36	23.5
	Upper Extremity (Shoulder-Arm-Forearm-Wrist-Hand)	19	12.4
	Lower Extremity (Hip-Thigh-Knee-Leg-Ankle-Foot)	7	4.6
	I have never suffered an injury	12	7.8
	Total	153	100.0
Frequency of Weekly Training	At least 3 days	102	66.7
	4–5 days	40	26.1
	6–7 days	11	7.2
	Total	153	100.0

**Table 2:** Validity and reliability results of the scales.

Variables	X^2^	df	CMIN/DF	GFI	AGFI	CFI	NFI	RMSEA	Cronbacha Alpha
			≤5	≥0.85	≥0.80	≥0.90	≥0.90	≤0.10	
1. Mental Training	142.48	36	3.95	0.91	0.87	0.94	0.90	0.08	0.78
2. Flow	115.42	48	2.40	0.88	0.89	0.92	0.90	0.06	0.81
3. Injury Anxiety	128.23	34	3.77	0.92	0.90	0.93	0.92	0.07	0.74

Note: Goodness of fit value ranges are arranged according to “acceptable standards”.

**Table 3:** Correlation analysis.

	N	X	SS	1	2	3
1. Mental Training	153	3.59	0.610	1		
2. Injury Anxiety	153	2.08	0.723	−0.277 **	1	
3. Flow	153	4.14	0.618	0.536 **	−0.514 **	1

Note: ** *p* < 0.01.

**Table 4:** The mediating role of athlete injury anxiety in the effect of mental training on flow.

Path Analysis		β	SE
Total Effect	(X → Y)	0.543 ***	0.066
Path a	(X → M)	−0.328 ***	0.088
Direct Effect	(X → Y)	0.432 ***	0.061
Path b	(M → Y)	−0.338 ***	0.052
Indirect Effect		0.111 **	0.034
**Model Fit Statistics**		**R^2^**	**F**
Total Effect Model		0.287	F_(1,167)_ = 67.24 ***
Path a Model		0.076	F_(1,167)_ = 13.90 ***
Full Mediation Model		0.432	F_(2,166)_ = 63.16 ***
**Bootstrap Results (5000 Samples)**			
Indirect Bootstrap Effect		(a.b) = 0.111 **	95% CI [0.044, 0.186]
The fully standardized effect size of mediation		(K^2^) = 0.109 **	95% CI [0.045, 0.176]

Note: X = Mental Training, M = Injury Anxiety, Y = Flow. Unstandardized coefficients reported. ** *p* < 0.01, *** *p* < 0.001.

## Data Availability

The raw data supporting the conclusions of this article will be made available by the authors upon request.

## Abbreviations

The following abbreviations are used in this manuscript:

MTS	Mental training scale
DOAJ	Sports injury anxiety scale
DFS-2	Dispositional flow scale
